# Disinfection and Disinfectants

**Published:** 1888-12

**Authors:** 


DISINFECTION AND DISINFECTANTS. Their application and use in the prevention and treatment of disease and in public and private sanitation, by the Committee on Disinfectants, appointed by the American Public Health Association. 1888: Irving A. Watson, Sec'y, Concord, N. H.

This book, just issued by the American Public Health Association, consists of the work of several distinguished American physicians appointed by the Association for the purpose of determining by experiment the value of different disinfectants in the prevention and treatment of disease. The following is a list of the authors: George M. Sternberg, M. D., Surgeon U. S. Army and Fellow by courtesy in the Johns Hopkins University; Joseph H. Raymond, M. D., Professor of Physiology and Sanitary Science in Long Island College Hospital; Victor C. Vaughan, M. D., Ph. D., Professor of Physiological Chemistry in the University of Michigan and member of the Michigan State Board of Health; Charles Smart, M. D., Surgeon U. S. Army and member of the National Board of Health; George H. Rohé, M. D., Professor of Hygiene in the College of Physicians and Surgeons, Baltimore; Joseph Holt, M. D., President of the Louisiana State Board of Health; Samuel H. Durgin, M. D., health officer of Boston; and J. R. Duggan, M. D. The work done by these specialists is of great importance and renders it the most complete and practical volume upon disinfection and disinfectants yet published. It consists of 265 pages with numerous illustrations, printed upon heavy paper, and is handsomely bound in cloth. The price has been placed at $2.00, postpaid, on receipt of the price.

